# Obesity, Thyroid Nodularity, and Thyroid Cancer: Epiphenomenon or Cause?

**DOI:** 10.1210/clinem/dgaa376

**Published:** 2020-06-11

**Authors:** David Bradley

**Affiliations:** Diabetes and Metabolism Research Center, Division of Endocrinology, Diabetes & Metabolism, Department of Internal Medicine, The Ohio State University, Columbus, OH

**Keywords:** obesity, type 2 diabetes, thyroid cancer, thyroid nodules, Mendelian randomization

Greater than two-thirds of the US population is overweight or obese, which not only increases the risk for type 2 diabetes (T2D) and components of the metabolic syndrome, but also appears to heighten the risk for numerous cancers including colorectal cancer, postmenopausal breast cancer, endometrial cancer, renal cell carcinoma, esophageal adenocarcinoma, pancreatic cancer, and liver cancer. More than half of all cancers diagnosed in women and nearly a quarter of all cancers diagnosed in men have been associated with being overweight and obese (1). Although the links between excess body weight and cancer risk are likely complex and potential mechanism(s) underlying this association are incompletely understood, many have been proposed including the coexistence of insulin resistance/hyperinsulinemia, abnormalities in hormone biosynthesis and hormonal pathways, disruption of circadian rhythm, and intestinal microbiome alterations, among others (2). In addition, obesity is associated with a chronic and systemic proinflammatory state in both adipose tissue and the local tumor microenvironment, which may be involved in a complicated cross-talk that propagates tumor development (3).

In particular, both observational and cohort studies have previously found a positive relationship between obesity, metabolic disease, and thyroid cancer. This association was illustrated by a recent systematic review of 15 studies that determined the link was not solely related to higher cancer detection (4) and a meta-analysis that reported a 25% greater risk of thyroid cancer in individuals who are overweight and a 55% greater thyroid cancer risk in individuals who are obese compared with individuals who are lean. Strikingly, a 5-unit increase in body mass index (BMI) and a 0.1-unit increase in waist to hip ratio increased the risk of thyroid cancer by 30% and 14%, respectively (5). This increased risk appeared to be specific for papillary, follicular, and anaplastic variants, with a reduced risk observed for medullary thyroid cancer, indicating a potential dependence on tumor type and histological specificity. These and other findings led the International Agency for Research on Cancer to conclude that thyroid cancer was one of 13 identifiable cancers in which obesity played a causal role, albeit conferring a small, but significant risk (odds ratio 1.17 in men and 1.04 in women) and was based largely on epidemiologic case-control and mechanistic studies (6).

The current epidemics of obesity and the metabolic/insulin resistance syndrome, which increase the risk of patients developing T2D, have also been accompanied by a rise in nodular thyroid disease, mainly in the form of nodular hyperplasia (7). Proposed mechanisms include a response to hyperinsulinemia, as insulin exerts both metabolic and mitogenic effects on target cells, and enhanced bioavailability of insulin-like growth factor-1 in concurrence with reduced hepatic insulin-like growth factor-binding protein 1 (8).

However, most studies to date have been limited by their cross-sectional, observational nature and difficulty in controlling for all potential confounding factors including age, gender, smoking status, history of radiation, and iodine intake. In addition, implicit bias may be introduced as patients who are obese are more likely to receive screening for not only thyroid function but thyroid nodularity, which may lead to an increase in thyroid cancer surveillance and, ultimately, diagnoses. Definitive evidence that weight loss interventions, including via either lifestyle or bariatric surgery, reduce the risk of thyroid cancer have also been mixed, with limited focus on potential mechanisms.

In the accompanying study by Fussey et al (9), the authors investigated whether excess adiposity and T2D are related to benign nodular thyroid disease and differentiated thyroid cancer in a large number of patients of European descent in the United Kingdom biobank, including 1812 patients who had benign nodules and 425 with differentiated thyroid cancer. They then further assessed causality by performing Mendelian randomization, an approach in which random genetic variants test whether traits such as obesity and T2D are causal to thyroid nodularity and cancer (and not the reverse), and do so without bias or many of the issues encountered with confounding factors. This type of analysis has not previously been reported for obesity and obesity-related comorbidities in relationship to either thyroid cancer or benign thyroid nodularity.

Their observational analyses found positive associations between benign nodular thyroid disease and both obesity and T2D (and a trend between obesity and thyroid cancer), largely consistent with the previous literature (4-6), even when controlling for age, sex, smoking status, alcohol consumption, Townsend deprivation index, BMI, and T2D. Yet through Mendelian randomization, surprisingly there was no observed causal role of obesity, and only a small increased odds ratio linking T2D and benign nodular thyroid disease and T2D and differentiated thyroid cancer in the highest quartile versus the lowest quartile of genetic liability. These results would suggest that obesity, in the absence of T2D, does not confer increased risk.

Although the findings are noteworthy, the study and its findings are not without limitations. The relatively low numbers of identified thyroid cancer and benign nodular cases may have been due to under self-reporting of diagnoses, undercoding, or exclusion of those with existing disease who did not undergo ultrasounds/biopsies, etcetera. Given the low number of patients with disease burden who were male, and the limited diversity of the patient population, the results may not be applicable to many groups of patients. In addition, the inclusion/exclusion criteria may have misclassified or excluded some of the participants, including those patients with type 1 diabetes who were diagnosed after the age of 35 years and had slow progression to insulin dependence, and patients with T2D who were excluded if they were started on insulin within a year of diagnosis. Lastly, the causal effect of obesity on medullary thyroid cancer cannot be determined.

Nonetheless, the study is an advancement in our understanding of the link between obesity, T2D, and thyroid disease and provides a valuable step in determining whether obesity (and related comorbidities) does, in fact, play a causal role in either benign nodular thyroid disease or differentiated thyroid cancer. There have been previous reports utilizing Mendelian randomization to assess causality with thyroid disease and atrial fibrillation and cardiovascular disease, yet this is the first report regarding obesity polymorphisms and the risk of thyroid nodules/thyroid cancer. Although not definitive due to several important limitations, including general applicability, the reported findings call into question whether the associations between obesity and differentiated thyroid cancer are simply an epiphenomenon and not a straightforward, identifiable cause-and-effect relationship.

Given the complexity of the topic, further investigation to replicate the findings in larger groups of patients, particularly in men and in those with different ethnic backgrounds, is needed. In addition, well-designed interventional studies assessing weight-loss effects on patient risk, with simultaneous mechanistic insight, will be invaluable toward further understanding and any therapeutic recommendations.

## Abbreviations

BMI: body mass index
T2D: type 2 diabetes

## Additional Information

***Disclosure Summary***: The author has nothing to disclose.

***Data Availability:*** Data sharing is not applicable to this article as no datasets were generated or analyzed during the current study.
